# Paediatric trainees and end-of-life care: a needs assessment for a formal educational intervention

**DOI:** 10.1007/s40037-015-0161-4

**Published:** 2015-02-03

**Authors:** Bonnie H Arzuaga, Leslie Caldarelli

**Affiliations:** 1 Department of Neonatology, Beth Israel Deaconess Medical Center, 330 Brookline Ave, RO 312, Boston, MA 02215 USA; 2 Department of Pediatrics, Harvard Medical School, Boston, MA USA; 3 Section of Neonatology, Department of Pediatrics, Comer Children’s Hospital, University of Chicago, Chicago, IL USA

**Keywords:** Graduate medical education, End of life care, Terminal care, Paediatrics

## Abstract

**Background:**

Literature suggests a paucity of formal training in end-of-life care in contemporary American medical education. Similar to trainees in adult medicine, paediatric trainees are frequently involved in end-of-life cases.

**Objective:**

To determine current experience and comfort levels among paediatric trainees when caring for dying patients with the hypothesis that more clinical experience alone would not improve comfort.

**Methods:**

Paediatric residents, subspeciality fellows and programme directors at the University of Chicago completed a voluntary electronic needs assessment in June and July 2013. Ten question pairs determined frequency of experiencing various aspects of end-of-life care in clinical practice and comfort levels during these encounters.

**Results:**

118 respondents participated (63.8 % response rate): 66.4 % were female; 53 % had previous education in end-of-life care. The proportion of those with experience in end-of-life care increased through the third year of training, and remained at 1.0 thereafter. Conversely, positive comfort scores increased gradually throughout all six years of training to a maximum proportion of 0.45. Comfort in many specific aspects of care lagged behind experience. Previous education had a significant positive effect on comfort levels of most, but not all, aspects of care. 58 % or more of trainees desired further education on specific end-of-life topics.

**Conclusions:**

Paediatric trainees are often involved in end-of-life care but may not be comfortable in this role. More experience alone does not improve comfort levels; however, there is a positive correlation with comfort and previous education. Trainees had a strong interest in further education on a variety of end-of-life care topics.

**Electronic supplementary material:**

The online version of this chapter (doi: 10.1007/s40037-015-0161-4) contains supplementary material, which is available to authorized users.

## Introduction

Literature suggests that physicians around the globe struggle with the many nuances of providing quality care to dying patients [1–5]. In the United States, medical education often lacks sufficient formal training in end-of-life care. An Association of American Medical Colleges (AAMC) review of medical school and teaching hospital curricula found them to be inadequate in areas of pain assessment, pain management, ethics, physician-patient communication, end-of-life communication, psychosocial care, personal awareness, bereavement and end-of-life clinical experiences [6]. The problem is pervasive, with trainees feeling unprepared to provide, and faculty feeling unprepared to teach, many of the key components of quality care for dying patients and their families [7, 8].

It has been shown that resident physicians gravitate towards subspecialties in which they feel most comfortable, demonstrating that increased comfort leads to greater perceived competence, which in turn dictates clinical practice [9]. This finding is supported by social cognitive theory, which describes how individuals’ choices and aspirations are influenced by their personal beliefs in self-efficacy [10]. Furthermore, learning areas in which residents report the least amount of comfort have also been the subjects they indicated to be in need of increased educational focus [9].

The Liaison Committee on Medical Education (LCME) required that clinical instruction include ‘the important aspects of…end-of-life care.’ [11]. In response, curricula templates for end-of-life care education have been published in family and internal medicine; however, these have invariably focused on issues applicable to adult patients, specifically geriatrics [12]. Similar to the trainees’ experiences in these fields, residents and fellows in paediatrics are frequently involved in end-of-life cases [13, 14] and attempts at developing educational initiatives have shown to be of some benefit [15, 16]. However, to date, there has been no standardized paediatric-based curriculum published.

The objective of this study was to determine both the current experiences and overall comfort levels among paediatric residents and subspeciality fellows when caring for a dying patient. The authors hypothesized that although trainees, the group inclusive of both residents and subspeciality fellows, may gain experience throughout their training, this alone will not correlate with improved levels of personal comfort when taking part in the medical management of children at the end-of-life.

## Methods

### Subject recruitment

In June and July 2013, an electronic needs assessment was distributed to all paediatric and medicine—paediatric residents, paediatric subspeciality fellows and paediatric programme directors at the University of Chicago Medicine Comer Children’s Hospital, an institution which lacks a standard paediatric end-of-life care curriculum. All postgraduate physician training programmes in the United States begin on 1 July of each year, and so by distributing in two separate time periods, the assessment was able to capture new medical school graduates (incoming PGY-1), interns finishing their first year of residency (finishing PGY-1), ongoing and graduating residents (finishing PGY-2 and PGY-3) as well as subspeciality fellows at all levels of training (finishing PGY-4 through PGY-6.)

### Survey design

The assessment included ten question pairs inquiring about personal experience with specific aspects of end-of-life care in blocks of increasing encounters (never experienced to > 20 encounters). Each aspect was derived from the inadequately covered areas reported in the AAMC review of teaching hospital curricula [6]. These included discussing a poor prognosis or giving ‘bad news;’ discussing goals of care with a patient and/or family; discussing advanced directives; discussing do not resuscitate orders; managing pain, nausea or fatigue medications for a dying patient; interacting with a family who insists on ‘doing everything’ despite a grim prognosis; discussing withdrawal of care with a family; being present during withdrawal of care; pronouncing death; and discussing autopsy options with the patient’s family following death. The second half of each pair was an inquiry about personal comfort. To measure comfort, respondents were required to rate statements beginning with ‘I feel comfortable…’ by choosing one of five responses on a Likert-scale: strongly disagree, disagree, unsure, agree, or strongly agree.

One follow-up question inquiring about the presence of effective personal coping mechanisms was included. A final question asked respondents to select from a list of end-of-life care topics those they felt could benefit from formal educational sessions. The assessment was anonymous and voluntary. Potential subjects received up to three weekly email reminders to complete the survey. The assessment is included in its entirety as an appendix to this article.

### Statistical analysis

Data was analyzed using STATA-12 (StataCorp LP, College Station, TX). The measured primary outcomes were clinical experience and comfort score. Ratings for the ‘comfort’ statements for each specific aspect were converted to a 5-point numerical scale with the answers of ‘strongly disagree’ and ‘strongly agree’ being equivalent to a score of one and five, respectively.

Proportion trends of both composite experience and comfort scores as well as experience and comfort for specific aspects of end-of-life care for all respondents from incoming PGY-1 to finishing PGY-6 were analyzed using chi-square test for trend. Experience was divided into ‘no exposure’ versus ‘exposure to one or more encounters.’ Comfort scoring was designated as ‘agree’ (scores of four or five on the Likert-scale), ‘unsure’ (score of three), or ‘disagree’ (scores of one or two). Composite scores for experience and comfort of finishing PGY-6 respondents and programme directors were compared separately using Fisher’s exact test.

In order to investigate the effect of previous end-of-life training on comfort scores, bivariate analysis using mean comfort scores with Fisher’s exact test followed by multivariate logistic regression including specific demographic variables.

Institutional Review Board exemption for this study was obtained prior to any data collection.

## Results

Of 185 eligible participants, 118 completed the assessment (63.8 % response rate). Table 1 shows participant demographics. Two-thirds of respondents were female and the mean age of the group was approximately 31 years. Overall, 53 % had exposure to formal end-of-life training in the past and, of those, 69 % indicated medical school as the setting for this training.

**Table 1:** Respondent demographics

Respondent demographics (*N* = 118)	*N* (%)
*Speciality/Subspeciality*	
Paediatric resident	58 (49.2)
Neonatal-perinatal medicine fellow	12 (10.2)
Medicine-paediatric resident	11 (9.3)
Paediatric critical care fellow	8 (6.8)
Developmental paediatrics fellow	6 (5.1)
Paediatric haematology-oncology fellow	4 (3.4)
Paediatric neurology fellow	3 (2.5)
Paediatric endocrinology fellow	3 (2.5)
Paediatric emergency medicine fellow	2 (1.7)
Paediatric infectious disease fellow	1 (0.8)
Paediatric gastroenterology fellow	1 (0.8)
Paediatric rheumatology fellow	1 (0.8)
*Year of training*	
Incoming PGY-1 (medical school graduates)	21 (18.4)
Finishing PGY-1	20 (17.5)
Finishing PGY-2	15 (13.2)
Finishing PGY-3 (residency programme graduates)	12 (10.5)
Finishing PGY-4	19 (16.7)
Finishing PGY-5	9 (7.9)
Finishing PGY-6	8 (7.0)
Paediatric Programme Directors	8 (6.8)
*Male*	37 (33.6)
*Female*	76 (66.4)
*Mean age (years)*	31.2 (range: 22–64)

**Fig. 1:**
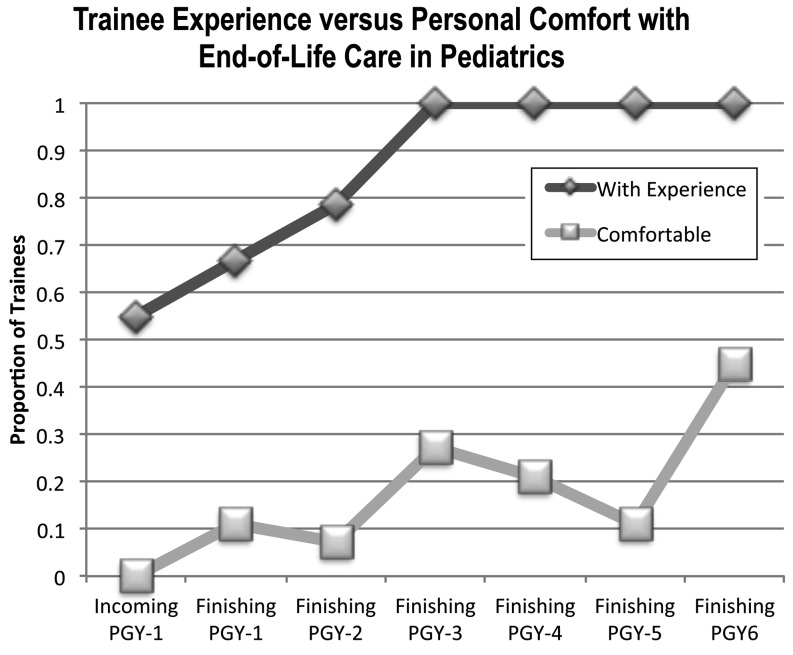
Trend analysis for proportions of experience and personal comfort scores by PGY level. ‘Comfort’ included those answers of ‘agree’ or ‘strongly agree’ to the statement ‘I feel comfortable….’ The proportion of those trainees with any experience begins at 0.55 for medical school graduates (incoming PGY-1) and reaches 1.0 by residency completion (finishing PGY-3) (*p* < 0.001). In contrast, those who report being comfortable begins at none, is 0.27 by residency completion, and reaches a maximum of 0.45 by fellowship completion (*p* = 0.005). Of note, overall proportions of those respondents agreeing that they are comfortable in end-of-life care are lower at all levels of training than the proportion of respondents who have actual experience with these circumstances

Figure 1 illustrates the trend analysis for overall experience with end-of-life encounters by PGY level compared with overall comfort with end-of-life care. The proportion of those with experience increased steadily from incoming PGY-1 to finishing PGY-2 and reached a plateau of 1.0 at finishing PGY-3 and beyond (*p* < 0.001). Contrary to this trend, those with a positive comfort score (defined as answers of ‘strongly agree’ or ‘agree’ with statements of feeling comfortable) showed a gradual increase throughout training years from incoming PGY-1 to finishing PGY-6 (*p* = 0.005). When finishing PGY-6 respondents were compared with programme directors, no difference was found in either the proportion of respondents with experience of at least ten encounters (*p *= 0.3) or in overall comfort scoring (*p* = 0.1).

**Fig.2:**
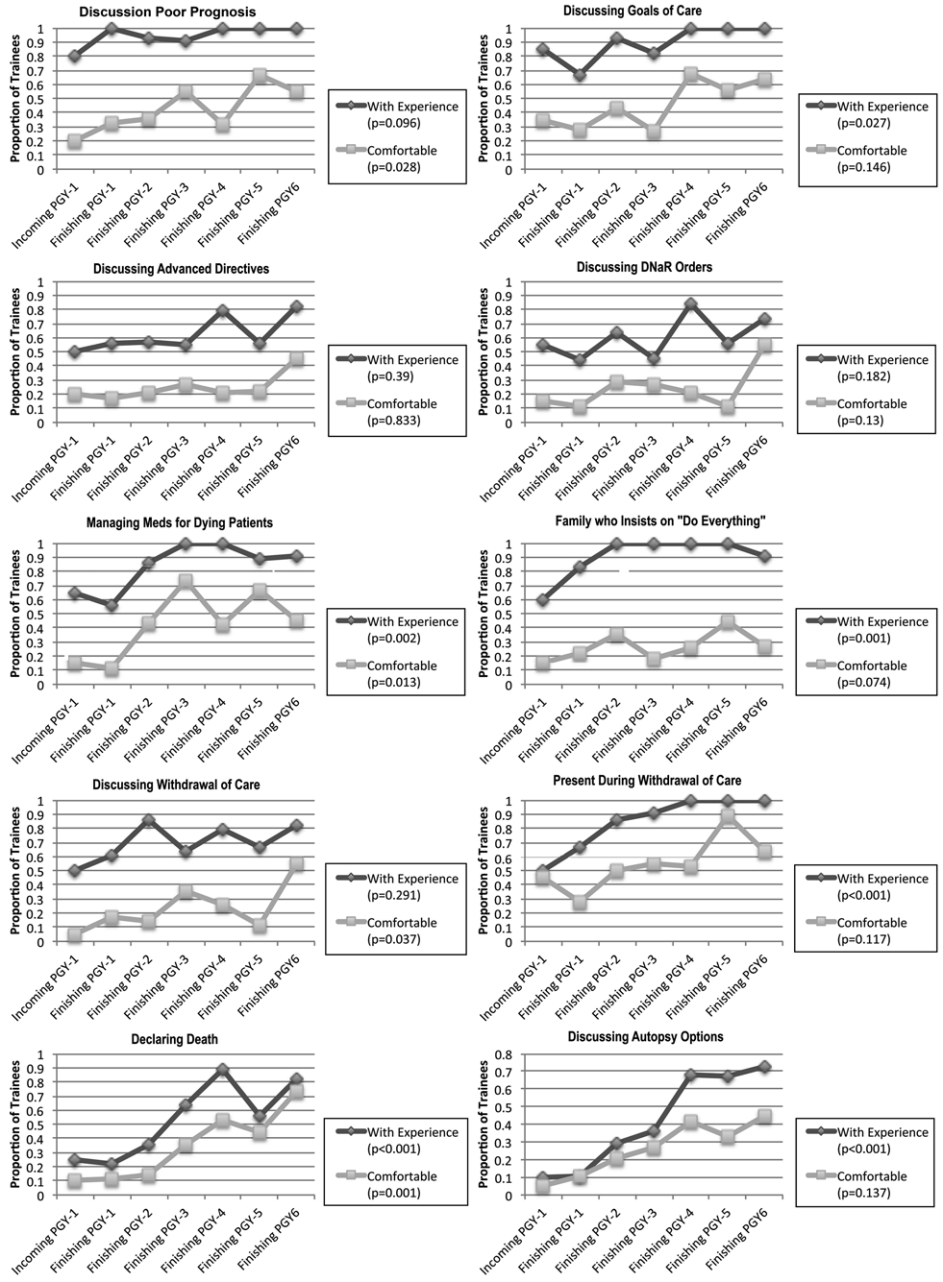
Trend analysis for proportions of experience and personal comfort scores for each specific aspect of end-of-life care queried in the assessment, by PGY level

The trend analyses of proportions of trainees with experience and who were comfortable with each specific aspect of end-of-life care, grouped by PGY level, are shown in Fig. 2. The aspects of care where experience, but not comfort, had a statistically significant increase during training were discussing goals of care (*p* = 0.027), discussing autopsy options (*p* < 0.001), being present during withdrawal of care (*p* < 0.001), and managing a patient whose family insists on ‘doing everything’ despite a grim prognosis (*p* = 0.01). Alternatively, the aspects of care where comfort, but not experience, increased significantly were discussing a poor prognosis (*p* = 0.028) and discussing withdrawal of care (*p* = 0.037). Aspects of care in which both experience and comfort increased over time included managing medications for dying patients and declaring death. Experience and comfort did not significantly increase for discussing advanced directives and discussing do-not-attempt resuscitation orders.

Previous exposure to formal end-of-life education created statistically significant improvements in mean comfort scores for discussing a poor prognosis (OR 3.6 [1.5, 8.5]; *p* = 0.003), goals of care (OR 3.4 [1.5, 7.7]; *p* = 0.004), advanced directives (OR 3.1 [1.3, 7]; *p* = 0.008), discussing withdrawal of care (OR 3.4 [1.5, 7.9]; *p* = 0.004) and autopsy options (OR 2.4 [1, 5.3]; *p* = 0.037), as well as managing pain medications (OR 2.6 [1.1, 6]; *p* = 0.023). Borderline improvements were noted in both discussing do-not-attempt resuscitation orders (OR 2.2 [0.99, 4.8]; *p* = 0.052) and in being present during withdrawal of care (OR 2.3 [0.99, 5.5]; *p* = 0.052). Previous training did not affect scores for declaring a patient dead (OR 1.4 [0.64, 3.2]; *p* = 0.88) or managing a difficult family who is insisting on ‘doing everything’ despite a grim prognosis (OR 1.5 [0.66, 3.5]; *p* = 0.32). When all measured aspects of end-of-life care were combined into an overall composite comfort score, 26.1 % of respondents with previous end-of-life care training had mean scores corresponding to ‘strongly agree’ or ‘agree’ versus 11.3 % of those without training (*p* = 0.079). Approximately half of respondents in both groups had mean composite comfort scores corresponding to an answer of ‘unsure’ in response to comfort. In multivariate analysis including age and PGY level, younger trainees were found to be significantly more comfortable than older trainees (*p* = 0.001) and they were also more likely to have received previous end-of-life training (*p* = 0.028). However, increase in PGY level nullified this effect.

In response to the question inquiring about physician bereavement, 65 % of respondents believed that they had effective personal coping mechanisms to deal with the death of a patient.

**Fig. 3:**
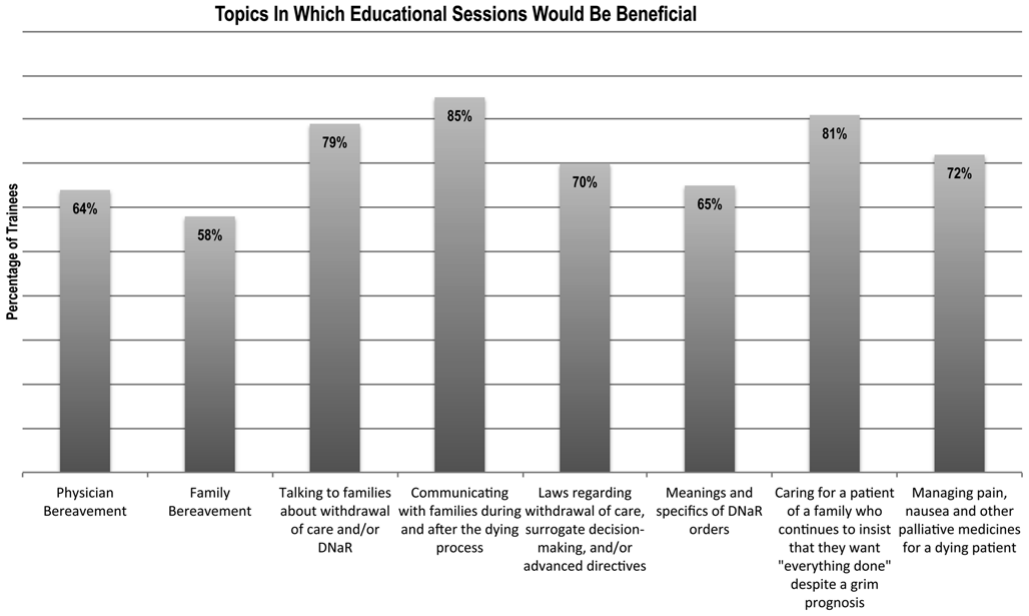
Self-reported educational needs by all trainees participating in the assessment. Trainees were asked to choose topics they felt they may benefit from attending sessions on or receiving more formal education in

Figure 3 shows percentages of trainees who indicated that they would benefit from attending an educational session covering a specific topic related to end-of-life care. Over half of the respondents were interested in attending sessions pertaining to all of the listed topics. The three most desirable topics were communicating with families during and after the dying process (85 %), caring for a patient whose family insists that ‘everything’ be done (81 %), and talking to families about withdrawal of care and/or do-not-attempt resuscitation orders (79 %).

## Discussion

This study demonstrates that paediatric trainees gain personal experience with end-of-life care during both residency and subspeciality fellowship. It also illustrates that trainees may not feel comfortable in this role and, importantly, that more clinical experience alone may not be enough to improve comfort levels. Rather, previous exposure to education in end-of-life care has a significant positive affect on comfort levels in many aspects of care for a dying patient and trainees have an overwhelming interest for more education related to these topics.

All of the trainees in this assessment had some experience caring for a dying child by the time they completed a three-year general paediatric residency programme. In terms of specific aspects of end-of-life care, increases in experience over the course of training, both in residency and subspeciality fellowship, were seen in almost all areas. For those aspects without statistically significant increases, the majority of participating trainees reported experience from the very beginning of paediatric training. Despite this, the proportion of those trainees who were comfortable with end-of-life care in paediatrics started at almost zero, increased very gradually, and did not reach over 40 % of respondents until the end of a subspeciality fellowship, or six years after medical school graduation. Further examination of specific aspects related to end-of-life care revealed that, while respondents may have greater improvement in comfort for some areas when compared with others, the proportions of those who report being comfortable falls below those with experience in all areas. Additionally, areas such as managing medications for dying patients and declaring death, where both experience and comfort increased in a similar fashion over time, were technical in nature rather than related to communication and the physician-patient or physician-family relationship.

Our data show that trainees completing their sixth year of subspeciality training do not differ significantly from programme directors in either experience or level of comfort in end-of-life care. This, in conjunction with the overall trend of a gradual increase in the proportion of trainees who are comfortable caring for a dying child, suggests that, in the current US postgraduate medical education environment, further training after residency improves physician comfort in end-of-life care. The lack of large increases in those who are comfortable with many aspects of end-of-life care until advanced training raises a concern that residents who do not receive additional fellowship education enter the workforce as primary care physicians who may not be significantly more comfortable in caring for a dying child then they were when they graduated from medical school. Primary care physicians may then be at a disadvantage as previous literature has shown that over the span of their careers the vast majority of general paediatricians will encounter a patient or patients who will die [17]. It can be postulated that those who go on to complete a subspeciality fellowship may have more opportunity to observe role models, participate in debriefings, or gain experience as a teacher to more junior physicians, all of which may aid in gaining comfort in caring for dying patients. Rather than mandating fellowship training or extending current residency training, more concentrated and focused education during the current residency years may be an alternative method designed to increase comfort in the early years of a paediatrician’s practice. This, in turn, can provide a sturdier foundation upon which comfort with end-of-life care can continue to evolve following completion of training. Further studies examining how end-of-life care changes with physician maturity as well as the additional factors that may influence this care are needed in order to better understand how more education early on may assist in future long-term career development.

There was an overwhelming positive response in this assessment to self-reported educational needs as they related to end-of-life care, which further supports the idea for more intensive end-of-life education during residency. Specifically, those topics related to communicating with families were the ones most desired by respondents for increased educational focus. A possibility for this may be inadequate coverage of these topics in previous training or may simply reflect the innate complexity of navigating physician-patient relationships with families of dying children.

Previous studies have shown that physicians’ behaviours are highly correlated with their perceived comfort and competence and therefore these markers may be indicative of their medical practices. One example of this is a study of medical students that showed the choice of pursuing a career in paediatrics was correlated with an above average score on a ‘death anxiety scale.’ [18] From an educational perspective, a resident who may have acquired the requisite knowledge and skills in end-of-life care may still be hindered by a lack of personal comfort [19]. Understanding of trainees’ obstacles for gaining comfort, such as personal values, religious beliefs, or concerns over legality, is critical in the development of a successful curriculum. International literature has demonstrated that factors such as these may be influential [3–5, 20] and future educational initiatives could focus on exploring these potential barriers.

In this study, 65 % of respondents believed that they had adequate personal mechanisms for coping with the deaths of their patients. It has been previously reported that 5–17.5 % of physicians experience moderate to severe intensity reactions to patient death and that those who do may benefit from increased supportive services [21, 22]. Younger physicians and medical students in particular are at risk for experiencing emotionally powerful reactions to deaths even when they were not close to the patients [23].

The current data represents a cross-sectional portrayal of paediatric trainees within one institution. Additionally, as the study utilized a survey method, the potential for response bias must be acknowledged. Despite these limitations, the current findings reflect themes that have been shown previously in the literature. Future studies conducted at more than one institution, and which inquire more deeply about the usefulness of previous training and/or specific experiences, may be helpful in teasing out both helpful factors and barriers against increased physician comfort in end-of-life situations.

## Conclusions

Paediatricians do not experience a reprieve from caring for dying patients despite their choice of speciality. While the factors influencing physician comfort with death are complex and likely multifactorial, a yearly increase in the number of clinical encounters with death may not in itself help young clinicians become more comfortable in caring for these patients and their families. Formal training that addresses the complexity of this topic in paediatric training programmes and development of appropriate curricula may be beneficial.

## Essentials

- Despite AAMC and LCME recommendations, almost half of the paediatric trainees in this study never experienced any formal education in end-of-life care prior to entering residency.
- Social cognitive theory suggests that perceived comfort correlates with perceived competence, which in turn dictates physician behaviour and clinical care.
- Paediatric residents are frequently involved in end-of-life care but more experience does not necessarily correlate to more comfort in providing this care.
- Increased comfort with many aspects of end-of-life care is positively affected by previous education and trainees show a high interest in receiving further education.
- Formal educational programmes in end-of-life care may have the potential to improve trainee comfort in the management of such patients and perhaps improve patient care.

## Electronic supplementary material

(PDF 38 kb)

## Abbreviations

PGY: Postgraduate year
