# Supplementary material for: Paediatric trainees and end-of-life care: a needs assessment for a formal educational intervention
Source: Perspect Med Educ. 2015 Feb 3;4(1):25–32. doi: 10.1007/s40037-015-0161-4 (PMC4348227; doi:10.1007/s40037-015-0161-4)
Supplement: Supplementary file 1 — (PDF 38 kb) [file 40037_2015_161_MOESM1_ESM.pdf]

End-of-Life Education

1. What type of practitioner are you?	
**Answer Options**	
Pediatric Resident	
Med-Peds Resident	
Ped Neurology Fellow	
Ped Critical Care Fellow	
Ped Pulmonology Fellow	
Ped Heme/Onc Fellow	
Ped Endocrinology Fellow	
Developmental Ped Fellow	
Neonatology Fellow	
Ped Gastroenterology Fellow	
Ped Emergency Medicine Fellow	
Ped Infectious Disease Fellow	
Ped Rheumatology Fellow	
General Pediatric Attending	
Pediatric Critical Care Attending (NICU or PICU)	
Pediatric Emergency Medicine Attending	
Pediatric Hematology/Oncology Attending	
Other Pediatric Subspecialty Attending	

2. What is your PGY level?	
**Answer Options**	
PGY-1 (start date June 2013)	
PGY-1	
PGY-2	
PGY-3	
PGY-4	
PGY-5	
PGY-6	
Attending Physician	
Other (please specify)	

3. Does your training program include any formal end-of-life educational curriculum?	
**Answer Options**	
Yes	

No	

4. How old are you (in years)?

Answer Options	

5. What is your gender?

Answer Options	
Female	
Male	

6. Have you had any previous formal training in end-of-life issues or related topics?

Answer Options	
Yes	
No	
If yes, where? (please specify)	

7. Have you ever had to discuss a poor prognosis (give “bad news”) to a patient and/or family?

Answer Options	
Yes, over 20 times	
10-20 times	
5-10 times	
1-5 times	
Never	

8. Rate how much you agree with the following statement: "I feel comfortable discussing a poor prognosis (giving “bad news”) to a patient and/or his family."

Answer Options	
Strongly Agree	
Agree	
Unsure	
Disagree	
Strongly Disagree	

9. Have you ever had to discuss “goals of care” with a patient and/or family?

Answer Options	
Yes, over 20 times	
10-20 times	
5-10 times	
1-5 times	
Never	

10. Rate how much you agree with the following statement: "I feel comfortable discussing “goals of care” with a patient and/or family."

Answer Options	
Strongly Agree	
Agree	
Unsure	
Disagree	
Strongly Disagree	

11. Have you ever had to discuss advance directives with a patient and/or family?

Answer Options	
Yes, over 20 times	
10-20 times	
5-10 times	
1-5 times	
Never	

12. Rate how much you agree with the following statement: "I feel comfortable discussing advance directives with a patient and/or family."	
**Answer Options**	
Strongly Agree<br>Agree<br>Unsure<br>Disagree<br>Strongly Disagree	

13. Have you ever had to discuss a do not resuscitate (DNR) order with the family of a dying patient?	
**Answer Options**	
Yes, over 20 times<br>10-20 times<br>5-10 times<br>1-5 times<br>Never	

14. Rate how much you agree with the following statement: "I feel comfortable discussing DNR orders with a patient and/or family."	
**Answer Options**	
Strongly Agree<br>Agree<br>Unsure<br>Disagree<br>Strongly Disagree	

15. Have you ever had to manage pain control, nausea and fatigue medications of dying patients?	
**Answer Options**	
Yes, over 20 times<br>10-20 times<br>5-10 times<br>1-5 times<br>Never	

16. Rate how much you agree with the following statement: "I feel comfortable managing pain control, nausea and fatigue medications of dying patients."

Answer Options	
Strongly Agree<br>Agree<br>Unsure<br>Disagree<br>Strongly Disagree	

17. Have you ever taken care of a dying patient whose family insisted on continuing to “do everything” despite a grim prognosis?

Answer Options	
Yes, over 20 times<br>10-20 times<br>5-10 times<br>1-5 times<br>Never	

18. Rate how much you agree with the following statement: "I feel comfortable taking care of a dying patient whose family insists on continuing to “do everything” despite a grim prognosis."

Answer Options	
Strongly agree<br>Agree<br>Unsure<br>Disagree<br>Strongly Disagree	

19. Have you ever had to discuss withdrawal of care with the family of a dying patient?

Answer Options	
Yes, over 20 times<br>10-20 times<br>5-10 times<br>1-5 times<br>Never	

20. Rate how much you agree with the following statement: "I feel comfortable discussing withdrawal of care with the family of a dying patient."

Answer Options	
Strongly Agree	
Agree	
Unsure	
Disagree	
Strongly Disagree	

21. Have you ever been present with a family during the withdrawal of care on a patient?

Answer Options	
Yes, over 20 times	
10-20 times	
5-10 times	
1-5 times	
Never	

22. Rate how much you agree with the following statement: "I feel comfortable being present with a family during withdrawal of care on a patient."

Answer Options	
Strongly Agree	
Agree	
Unsure	
Disagree	
Strongly Disagree	

23. Have you ever declared a patient dead?

Answer Options	
Yes, over 20 times	
10-20 times	
5-10 times	
1-5 times	
Never	

24. Rate how much you agree with the following statement: "I feel comfortable declaring a patient dead."

Answer Options	
Strongly Agree	
Agree	
Unsure	
Disagree	
Strongly Disagree	

25. Have you ever had to discuss autopsy options with a family?

Answer Options	
Yes, over 20 times	
10-20 times	
5-10 times	
1-5 times	
Never	

26. Rate how much you agree with the following statement: "I feel comfortable discussing autopsy options with a family."

Answer Options	
Strongly Agree	
Agree	
Unsure	
Disagree	
Strongly Disagree	

27. Do you feel that you have effective personal coping mechanisms to deal with the death of your patients?

Answer Options	
Yes	
No	
If yes, can you give some examples of how you handle deaths?	

28. Do you feel that there are effective hospital-based support services to help clinicians deal with the death of their patients?

Answer Options
Yes
No

29. What are some areas that you feel you may benefit from either attending sessions on or having more formal education in? (check all that apply)

Answer Options
Physician Bereavement
Family Bereavement
Talking to families about withdrawal of care and/or DNR
Communicating with families during and after the dying
Laws regarding withdrawal of care, surrogate decision-
Meanings and specifics of DNR orders
Dealing with the family who continues to insist that they want
Managing pain, nausea, fatigue and other palliative
Other Suggestions (please specify)
